# Development of a Cell-Based Luciferase Complementation Assay for Identification of SARS-CoV-2 3CL^pro^ Inhibitors

**DOI:** 10.3390/v13020173

**Published:** 2021-01-24

**Authors:** Jonathan M. O. Rawson, Alice Duchon, Olga A. Nikolaitchik, Vinay K. Pathak, Wei-Shau Hu

**Affiliations:** 1 Viral Recombination Section, HIV Dynamics and Replication Program, National Cancer Institute, Frederick, MD 21702, USA; jonathan.rawson@nih.gov (J.M.O.R.); alice.duchon@nih.gov (A.D.); nikolaio@mail.nih.gov (O.A.N.); 2 Viral Mutation Section, HIV Dynamics and Replication Program, National Cancer Institute, Frederick, MD 21702, USA; pathakv@mail.nih.gov

**Keywords:** SARS-CoV-2, COVID-19, 3CL^pro^, protease, luciferase, antiviral, inhibitor

## Abstract

The 3C-like protease (3CL^pro^) of SARS-CoV-2 is considered an excellent target for COVID-19 antiviral drug development because it is essential for viral replication and has a cleavage specificity distinct from human proteases. However, drug development for 3CL^pro^ has been hindered by a lack of cell-based reporter assays that can be performed in a BSL-2 setting. Current efforts to identify 3CL^pro^ inhibitors largely rely upon in vitro screening, which fails to account for cell permeability and cytotoxicity of compounds, or assays involving replication-competent virus, which must be performed in a BSL-3 facility. To address these limitations, we have developed a novel cell-based luciferase complementation reporter assay to identify inhibitors of SARS-CoV-2 3CL^pro^ in a BSL-2 setting. The assay is based on a lentiviral vector that co-expresses 3CL^pro^ and two luciferase fragments linked together by a 3CL^pro^ cleavage site. 3CL^pro^-mediated cleavage results in a loss of complementation and low luciferase activity, whereas inhibition of 3CL^pro^ results in 10-fold higher levels of luciferase activity. The luciferase reporter assay can easily distinguish true 3CL^pro^ inhibition from cytotoxicity, a powerful feature that should reduce false positives during screening. Using the assay, we screened 32 small molecules for activity against SARS-CoV-2 3CL^pro^, including HIV protease inhibitors, HCV protease inhibitors, and various other compounds that have been reported to inhibit SARS-CoV-2 3CL^pro^. Of these, only five exhibited significant inhibition of 3CL^pro^ in cells: GC376, boceprevir, Z-FA-FMK, calpain inhibitor XII, and GRL-0496. This assay should greatly facilitate efforts to identify more potent inhibitors of SARS-CoV-2 3CL^pro^.

## 1. Introduction

In December 2019, a novel human coronavirus, now known as severe acute respiratory syndrome coronavirus 2 (SARS-CoV-2), emerged in Wuhan, China [1,2]. This virus is the causative agent of the global COVID-19 pandemic, which has resulted in over 70 million infections and 1.5 million deaths as of 11 December 2020. While promising results have been reported for several candidate vaccines in clinical trials [3,4,5], the widespread distribution of any vaccine will require a significant amount of time, leading to a pressing need for antiviral therapeutics. Recently, two monoclonal antibody therapies received emergency use authorization from the FDA for treatment of mild-to-moderate COVID-19 in patients at high risk for severe disease [6]. In addition, remdesivir, a nucleotide analog that inhibits the SARS-CoV-2 RNA-dependent RNA polymerase [7], has been approved by the FDA for COVID-19 in hospitalized patients. However, clinical trials of remdesivir have produced mixed results [8,9,10,11], highlighting an urgent need for additional antiviral drugs.

Following entry and uncoating of the SARS-CoV-2 genome into the cytoplasm, two overlapping polyproteins, pp1a and pp1ab, are expressed by translation of ORF1a and 1b, with the latter resulting from −1 programmed ribosomal frameshifting [12]. These polyproteins contain two cysteine proteases: papain-like protease (PL^pro^, Nsp3) and 3C-like protease (3CL^pro^, Mpro, Nsp5), which are responsible for 3 and 11 cleavage events, respectively [12]. SARS-CoV-2 3CL^pro^ consists of three domains: Domain I (residues 10–99) and II (residues 100–182) resemble picornavirus 3C proteases and chymotrypsin [13]. The active site, which contains a Cys-His catalytic dyad (H41, C145), is situated between these domains. Domain III (residues 198–303) regulates 3CL^pro^ homodimerization, which is required for enzymatic activity [14]. 3CL^pro^ is widely regarded as an excellent target for COVID-19 drug development because it is essential for viral replication and has a cleavage specificity distinct from human cellular proteases, reducing the likelihood of off-target effects [13,15,16]. The preferred sequence at cleavage sites is LQ ↓ (S, A, G), with the arrow indicating the cleavage site [13]. Furthermore, the structure of 3CL^pro^ is highly conserved across coronaviruses, suggesting it may be possible to develop pan-coronavirus inhibitors that target 3CL^pro^ [17].

Unfortunately, drug development for SARS-CoV-2 3CL^pro^ has been greatly hindered by a lack of tractable, cell-based assays that can be performed in a biosafety level 2 (BSL-2) setting. Some of the existing experimental methods that have been used to identify 3CL^pro^ inhibitors are described below. Many groups have performed in vitro screens using purified 3CL^pro^ protein [18,19,20,21,22,23,24], but these systems do not account for cell permeability, metabolization, or cytotoxicity of compounds. In addition, the recombinant 3CL^pro^ protein used in these assays may differ from 3CL^pro^ expressed in human cells. Consequently, initial hits often have high toxicity and/or poor activity in cell-based assays. Alternatively, inhibitors can be identified by directly assessing SARS-CoV-2 replication in cell culture, but this requires biosafety level 3 (BSL-3) containment and is not amenable to high-throughput screening. Additionally, in these assays, virus replication is often quantified by a plaque assay or quantitative RT-PCR, which can lead to difficulties in distinguishing antiviral activity from cytotoxicity, as cytotoxicity also results in reduced virus production. Recently, a cell-based BSL-2 reporter system for identification of SARS-CoV-2 3CL^pro^ inhibitors was developed [25]. However, this system, based on a fluorescent reporter called Flip-GFP, represents a loss-of-function approach, which may lead to difficulty in discriminating cytotoxicity from 3CL^pro^ inhibition. Lastly, virtual screening and molecular dynamics simulations have widely been used to identify candidate inhibitors for experimental follow-up [26,27,28].

Here, we describe a novel luciferase complementation reporter assay to identify inhibitors of SARS-CoV-2 3CL^pro^ in living cells. The assay can be performed in a BSL-2 setting and is based on a single lentiviral vector that co-expresses 3CL^pro^ and a split reporter in which two luciferase fragments are connected by a cleavage site. When 3CL^pro^ cuts the cleavage site, luciferase complementation is lost, resulting in low luciferase activity. In contrast, when a compound is added that inhibits 3CL^pro^, cleavage is blocked and complementation is maintained, resulting in significantly higher luciferase activity. Using the assay, we screened 32 small molecules for activity against SARS-CoV-2 3CL^pro^, leading to the identification of five active compounds: GC376, boceprevir, Z-FA-FMK, calpain inhibitor XII, and GRL-0496. The luciferase reporter assay is sensitive, rapid, easy to perform, and can readily differentiate cytotoxicity from true inhibition of 3CL^pro^, a feature that should be advantageous during antiviral drug screening.

## 2. Materials and Methods

### 2.1. Plasmids, Cell Lines, and Reagents

Lentiviral plasmids encoding human codon-optimized SARS-CoV-2 3CL^pro^ (wild-type [WT] or C145A) were generous gifts from Nevan Korgan (pLVX-EF1α-SARS-CoV-2-nsp5-2xStrep-IRES-Puro, Addgene # 141370 [WT] and # 141371 [C145A]) [29]. NanoBiT plasmids encoding SmBiT (S) or LgBiT (L) were purchased from Promega (Madison, WI, USA). The 2xStrep tag on the C-terminal end of 3CL^pro^ was replaced with P2A from porcine teschovirus-1 (GSGATNFSLLKQAGDVEENPGP), S, L, and green fluorescent protein (GFP) using NdeI and BamHI restriction sites. S and L were separated by two flexible linkers (each with the sequence GGGSGGGSGGGS) surrounding a SARS-CoV-2 nsp4-nsp5 cleavage site (TSAVLQ↓SGFRKM, with the arrow indicating the cleavage site). 293T human embryonic kidney cells were obtained from American Type Culture Collection (Manassas, VA, USA; catalog # CRL-3216) and maintained in Dulbecco’s modified Eagle’s medium supplemented with 10% fetal bovine serum (FBS), 100 U/mL penicillin, and 100 µg/mL streptomycin. GC376, boceprevir, telaprevir, simeprevir, asunaprevir, grazoprevir, ebselen, Z-FA-FMK, TBB, masitinib, and doxorubicin were obtained from Selleck Chemicals (Houston, TX, USA). Calpain inhibitor XII, walrycin B, DA-3003-1, MG-115, suramin, and quinacrine were obtained from Cayman Chemical (Ann Arbor, MI, USA). Calpain inhibitor II, penta-O-galloyl-beta-D-glucose hydrate, baicalein, and tafenoquine were obtained from Sigma (St. Louis, MO, USA). GRL-0496 was obtained from Focus Biomolecules (Plymouth Meeting, PA, USA). MK 0893 was obtained from APExBIO (Houston, TX, USA), and MAC 5576 was obtained from Thermo Fisher Scientific (Waltham, MA, USA). HIV protease inhibitors were obtained from the NIH AIDS Reagent Program, Division of AIDS, NIAID, NIH (Bethesda, MD, USA).

### 2.2. Transfections and Luciferase Assays

For comparison of luciferase reporter constructs, 293T cells were seeded into solid white 96-well plates, using 10,000 cells in 0.1 mL medium per well. Cells were transfected 24 h later with 100 ng or 20 ng 3CL^pro^ plasmid, using 0.25 µL TransIT-LT1/well (Mirus Bio; Madison, WI, USA). For the transfections of 20 ng plasmid, 80 ng carrier DNA (Promega; Madison, WI, USA) was included to normalize the total amount of DNA. Dimethyl sulfoxide (DMSO) or 100 µM GC376 was added 4 h post-transfection. Luciferase activity was measured 30 h post-transfection using the Nano-Glo Live Cell Assay System (Promega), following the manufacturer’s instructions. Briefly, the 96-well plates, Opti-Mem I reduced serum medium (Life Technologies; Carlsbad, CA, USA), Nano-Glo dilution buffer, and Nano-Glo live cell substrate were equilibrated to room temperature for 30 min. Next, the medium on the cells was aspirated and replaced with 100 µL Opti-Mem I, 23.75 µL Nano-Glo dilution buffer, and 1.25 µL Nano-Glo live cell substrate/well. The plate was mixed on an orbital shaker for 15 s and immediately read using a SpectraMax iD3 (Molecular Devices; San Jose, CA, USA) and an integration time of 400 ms. Compounds were tested for inhibition of 3CL^pro^ following a similar protocol with several small modifications. The 293T cells were seeded into 6-well plates, using 330,000 cells in 2 mL medium per well. Cells were transfected 24 h later with 2 µg 3CL^pro^ plasmid and 5 µL TransIT-LT1 per well. Cells were seeded 4 h post-transfection into solid white 96-well plates, using 10,000 cells in 0.1 mL medium per well. Compounds (or DMSO) were immediately added to final concentrations ranging from 0.1 to 100 µM. Untransfected cells with DMSO, transfected cells with DMSO, and transfected cells with 100 µM GC376 were included as controls on every plate. Luciferase activity was measured 30 h post-transfection as described. The average luciferase activity of transfected cells with DMSO was subtracted from the values obtained for the other samples, and the data were further normalized to the value obtained for transfected cells with 100 µM GC376, which was set to 100%. NanoBiT data for the DMSO control were graphed at a drug concentration of 10 nM so that they could be displayed on a log axis.

### 2.3. Cell Viability Analysis

Cell viability was measured using the CellTiter-Glo 2.0 cell viability assay (Promega), following the manufacturer’s instructions. Briefly, 293T cells were seeded, transfected, and treated with compounds as described for the NanoBiT luciferase assay. Cell viability was measured 30 h post-transfection by equilibrating the 96-well plate and CellTiter-Glo 2.0 reagent to room temperature for 30 min and adding 100 µL reagent per well. The plate was mixed for 2 min on an orbital shaker, and firefly luciferase activity was measured 10 min later using a SpectraMax iD3 (Molecular Devices) and an integration time of 400 ms. The average luciferase activity in wells containing only media was subtracted from the values obtained for the other samples, and the data were further normalized to the value obtained for transfected cells with DMSO, which was set to 100%.

### 2.4. Western Blotting

The 293T cells were seeded into 6-well plates, using 330,000 cells in 2 mL medium per well. Cells were transfected 24 h later with 2 or 0.4 µg 3CL^pro^ plasmid and 5 µL TransIT-LT1/well (Mirus Bio). For the transfections of 0.4 µg plasmid, 1.6 µg carrier DNA (Promega) was included to normalize the total amount of DNA. DMSO or 100 µM GC376 was added 4 h post-transfection. At 30 h post-transfection, the cells were washed with phosphate-buffered saline and lysed on plate using CelLytic solution (Sigma) containing cOmplete EDTA-free protease inhibitor cocktail tablets (Roche; Basel, Switzerland). Cell lysates were centrifuged at 16,000× *g* for 10 min at 4 °C, and supernatants were transferred to new tubes. Lastly, an appropriate volume of 4× Laemmli sample buffer (Bio-Rad; Hercules, CA, USA) containing 10% beta-mercaptoethanol was added to cell lysates, and the samples were incubated at 99 °C for 5 min. SDS-PAGE was performed using 4–20% Criterion TGX precast gels (Bio-Rad). After transfer to PVDF membranes, blots were probed with 1:5000 rabbit anti-SARS-CoV 3CL^pro^ (Rockland Immunochemicals; Limerick, PA, USA; catalog #200-401-A51), mouse anti-P2A (Novus Biologicals; Littleton, CO, USA; catalog #NBP2-59627), mouse anti-L (Promega; catalog #N710A), or rabbit anti-GFP antibodies (Invitrogen; Carlsbad, CA, USA; catalog #A6455). These were followed with incubation of either goat anti-mouse IgG (IRDye 680CW; LI-COR; Lincoln, NE, USA) or goat anti-rabbit IgG (IRDye 800RD, LI-COR) secondary antibodies at 1:10,000 dilutions. Blots were also probed with mouse anti-HSP90 antibody (Santa Cruz Biotechnology; Dallas, TX, USA; catalog #SC-69703) at a dilution of 1:10,000 as a loading control. Western blots were imaged and quantified using the Odyssey CLx infrared imaging system and Image Studio Lite v5.2 (LI-COR). To calculate the percentage of uncleaved S-L-GFP reporter, background subtracted intensities of S-L-GFP bands were divided by the sum intensities of S-L-GFP and L-GFP bands. For each experiment, at least three independent blots were performed.

### 2.5. Statistical Analysis

Statistical analysis was performed in GraphPad Prism v8.4.3 (GraphPad Software, San Diego, CA, USA). In the first figure, data were analyzed by mixed-effects analysis, followed by Dunnett’s post-test and correction for multiple comparisons. The treatment and vector type were considered fixed effects, whereas the biological replicate was considered a random effect. In the remaining figures, data were analyzed by one-way ANOVA followed by Dunnett’s post-test and correction for multiple comparisons. For all experiments, at least three independent biological replicates were performed.

## 3. Results

### 3.1. Development of Cell-Based Luciferase Complementation Reporters to Detect Inhibition of SARS-CoV-2 3CL^pro^ Activity

**Figure 1:**
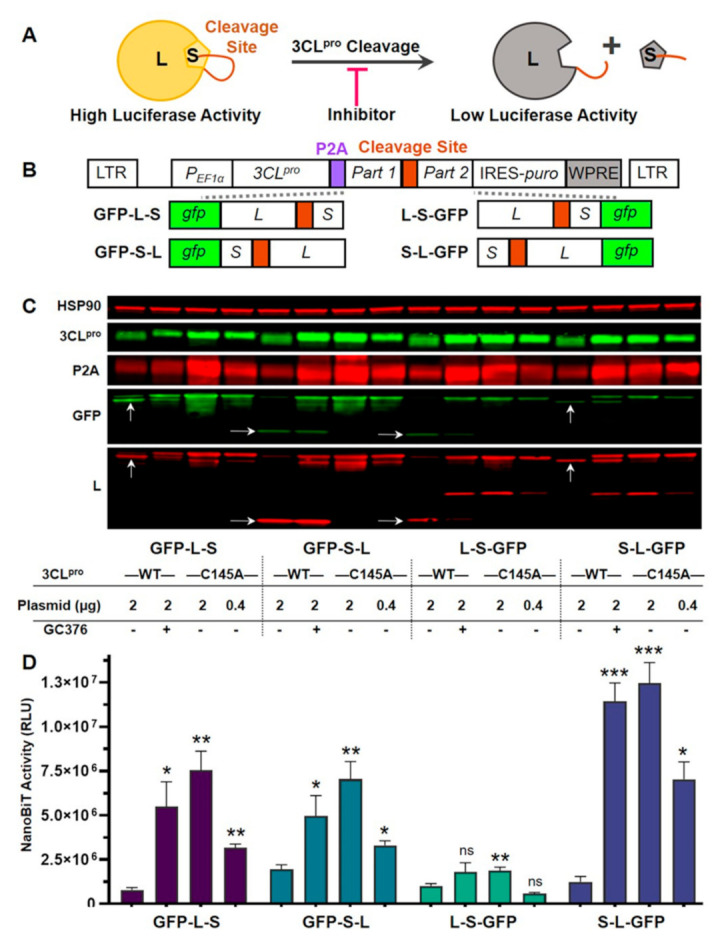
Development of cell-based luciferase complementation reporters to detect inhibition of SARS-CoV-2 3CL^pro^ activity. (**A**) Strategy for detection of 3CL^pro^ inhibition using NanoBiT, a luciferase complementation reporter comprised of Large BiT (L) and Small BiT (S). When connected by a linker containing a 3CL^pro^ cleavage site, 3CL^pro^ cleavage should result in a loss of complementation and low luciferase activity. Conversely, 3CL^pro^ inhibitors should prevent cleavage, resulting in high luciferase activity. (**B**) Lentiviral vectors that express 3CL^pro^ and reporters. P2A, a self-cleaving peptide, was inserted between 3CL^pro^ and reporters. Reporters consisted of GFP linked to L and S in various orders. L and S were separated by the SARS-CoV-2 nsp4-nsp5 cleavage site. LTR, long terminal repeat; P_EF1α_, EF1α promoter; IRES, internal ribosome entry site; puro, puromycin resistance gene; WPRE, woodchuck hepatitis virus post-transcriptional regulatory element. (**C**) Western blotting to examine reporter expression and cleavage. Lentiviral vectors expressing 3CL^pro^ WT or C145A (a catalytically inactive mutant) were transfected into 293T cells, and DMSO (control) or the 3CL^pro^ inhibitor GC376 (100 µM) was added. Cell lysates were collected 30 h post-transfection. 3CL^pro^ was detected using anti-SARS-CoV 3CL^pro^ or anti-P2A antibodies, whereas reporters were detected using anti-GFP or anti-L antibodies. Arrows denote cleavage products of expected sizes. For 3CL^pro^ C145A, two different plasmid amounts were transfected: 2 or 0.4 µg. HSP90 was included as a loading control. (**D**) NanoBiT luciferase activity. 293T cells were transfected (same sample order as in **C**), and NanoBiT activity (expressed as relative luciferase units, or RLU) was measured 30 h post-transfection. The data represent the mean ± standard deviation of four independent experiments; ns: not significant; * *p* < 0.05, ** *p* < 0.01, *** *p* < 0.001 (relative to 3CL^pro^ WT without drug; mixed effects analysis with Dunnett’s post-test).

To develop a reporter for inhibition of SARS-CoV-2 3CL^pro^ activity, we chose to use NanoBiT, a complementation reporter engineered from NanoLuc luciferase [30]. NanoBiT is comprised of two protein fragments: Large BiT (L, 17.6 kDa) and Small BiT (S,1.3 kDa). When the L and S fragments are in close proximity, they can complement each other to form a functional luciferase protein. However, when the two fragments are not in close proximity, they do not associate, leading to low luciferase activity. Importantly, this system was engineered to be fully reversible. We connected the L and S fragments with a linker containing the SARS-CoV-2 3CL^pro^ nsp4-nsp5 cleavage site (TSAVLQ↓SGFRKM, with the arrow indicating the cleavage site) (Figure 1A). We hypothesized that when 3CL^pro^ is expressed, it would cleave the linker, causing the two NanoBiT fragments to dissociate and resulting in low luciferase activity. In contrast, when 3CL^pro^ is inhibited (e.g., by a small molecule) or inactivated (e.g., by mutation of its catalytic site), NanoBiT would remain intact, leading to high luciferase activity. Thus, 3CL^pro^ inhibition should result in a gain, rather than a loss, of luciferase reporter signal. In principle, this feature would also allow the discrimination of 3CL^pro^ inhibition from cytotoxicity, thereby avoiding false positives from cytotoxic compounds.

To test this strategy, we constructed lentiviral vectors that encode both SARS-CoV-2 3CL^pro^ and reporter cassettes (Figure 1B). Four different reporter cassettes were designed by fusing green fluorescent protein (GFP), L, and S in various orders. GFP was included as a marker for transfection and expression; previous studies have indicated that both GFP and NanoLuc luciferase remain functional when fused together [31,32]. L and S were separated by a nsp4-nsp5 cleavage site surrounded by two flexible linkers. 3CL^pro^ and the reporter cassette were separated by P2A, a small “self-cleaving” peptide from porcine teschovirus-1 [33]. After cleavage, most of the P2A sequence should remain on the C-terminus of 3CL^pro^; a previous report indicated that SARS-CoV-2 3CL^pro^ maintains high activity when tagged on the C-terminus [20]. These lentiviral vectors should express equimolar amounts of 3CL^pro^ and reporter. In addition to vectors encoding wild-type (WT) 3CL^pro^, we also constructed four control vectors encoding 3CL^pro^ C145A, a catalytically inactive mutant [29].

To analyze expression and cleavage, the lentiviral vectors were transfected into 293T cells, followed by the addition of DMSO (control) or 100 µM GC376, a small molecule inhibitor of SARS-CoV-2 3CL^pro^ [18,19,20,34]. GFP was clearly detectable by fluorescence microscopy and flow cytometry in cells transfected with each vector (data not shown), indicating that GFP remained functional when fused to L and S. Western blotting was performed on cell lysates collected 30 h post-transfection (Figure 1C). Expression of 3CL^pro^ and reporters was similar between vectors with different reporter cassettes. However, inhibition or inactivation of 3CL^pro^ with GC376 or the C145A mutation, respectively, resulted in increased expression of both 3CL^pro^ and reporters. Others have also observed that 3CL^pro^ C145A is expressed better than 3CL^pro^ WT [29]. In the absence of GC376, 3CL^pro^ WT cleaved each GFP-NanoBiT reporter, generating products of the expected sizes (arrows in Figure 1C). As expected, these cleavage products were not detected with 3CL^pro^ C145A. In the presence of GC376, cleavage by 3CL^pro^ WT was partially prevented, generating an intermediate phenotype.

To determine if 3CL^pro^ inhibition led to increased luciferase activity, we measured NanoBiT luciferase activity in transfected 293T cells (Figure 1D). For 3 out of 4 reporter cassettes (all except L-S-GFP), compared to the 3CL^pro^ WT control, we observed significant increases in luciferase activity for both 3CL^pro^ WT with GC376 and 3CL^pro^ C145A. The lentiviral vector with the S-L-GFP reporter cassette had the greatest enhancement of luciferase activity for both 3CL^pro^ WT with GC376 (9.7-fold difference) and 3CL^pro^ C145A (10.6-fold difference). To determine if increased luciferase activity was due to higher levels of protein expression, we also transfected five-fold less of the 3CL^pro^ C145A vector DNAs, which resulted in comparable protein expression between 3CL^pro^ WT and C145A vectors (Figure 1C). For three out of four reporter cassettes (all except L-S-GFP), we observed significantly higher luciferase activity for 3CL^pro^ C145A than for 3CL^pro^ WT. Thus, 3CL^pro^ inhibition led to enhanced luciferase activity due to the combined effects of increased expression and reduced 3CL^pro^-mediated cleavage. As the lentiviral vector with the S-L-GFP reporter cassette had the best signal-to-background ratio, this vector was used for all further analysis. 

### 3.2. The Luciferase Complementation Assay Accurately Reflects Concentration-Dependent Inhibition of SARS-CoV-2 3CL^pro^-Mediated Cleavage

**Figure 2:**
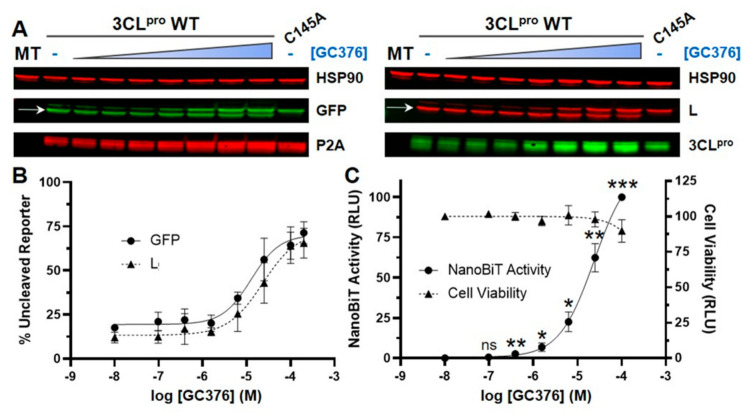
The luciferase complementation assay accurately reflects concentration-dependent inhibition of SARS-CoV-2 3CL^pro^-mediated cleavage. (**A**) Western blotting to examine reporter cleavage. 293T cells were transfected with the lentiviral vector containing 3CL^pro^ WT and the S-L-GFP reporter cassette, and DMSO (control) or GC376 (0.1, 0.4, 1.6, 6.3, 25, 100, or 200 µM) was added. Mock transfection (MT) and 3CL^pro^ C145A S-L-GFP were included as controls. On the GFP and LgBiT (L) panels, the bottom band corresponds to cleaved reporter (L-GFP, 46 kDa, indicated by arrows), while the top band corresponds to uncleaved reporter (S-L-GFP, 49 kDa). (**B**) Quantification of the percentage of uncleaved reporter from three independent western blots based on GFP (circle) or L (triangle) detection. (**C**) NanoBiT luciferase activity and cell viability. 293T cells were transfected with the S-L-GFP lentiviral vector, and DMSO (control) or GC376 (0.1, 0.4, 1.6, 6.3, 25, or 100 µM) was added. NanoBiT activity (circle, left axis) and cell viability (measured with the CellTiter-Glo 2.0 assay; triangle, right axis) were analyzed 30 h post-transfection and are expressed as relative luciferase units (RLU). Cell viability data were normalized to the DMSO sample, which was set to 100%. NanoBiT data were normalized to the DMSO and 100 µM GC376 samples, which were set to 0 and 100%, respectively. The data represent the mean ± standard deviation of four independent experiments; ns: not significant; * *p* < 0.05, ** *p* < 0.01, *** *p* < 0.001 (relative to no-drug; one-way ANOVA with Dunnett’s post-test).

To evaluate whether the luciferase complementation assay reflects the concentration-dependent inhibition of 3CL^pro^-mediated cleavage, we treated cells transfected with the S-L-GFP lentiviral vector with varying concentrations of GC376 (ranging from 0.1 to 200 µM). By Western blotting, we found that only 15% of the S-L-GFP reporter remained uncleaved in the absence of GC376 (Figure 2A, lane 2). In the presence of GC376, we observed concentration-dependent inhibition of 3CL^pro^-mediated cleavage, with half maximal effective concentration (EC_50_) values of 12.6 µM as detected by anti-GFP antibody or 23.2 µM as detected by anti-L antibody (Figure 2B). Maximally (i.e., at 200 µM), GC376 prevented cleavage of ~70% of the S-L-GFP reporter. We next determined the effects of varying concentrations of GC376 on luciferase reporter activity and cell viability (Figure 2C). GC376 resulted in a concentration-dependent increase in luciferase activity, with an EC_50_ of 23.8 µM, which is close to the values obtained by Western blotting. The increased luciferase activity observed with GC376 was statistically significant (relative to no drug) at all concentrations tested except for the lowest concentration (0.1 µM). We also found that GC376 had no significant effect on cell viability up to 100 µM (Figure 2C). These data further confirm the validity of the luciferase complementation assay for detecting inhibition of SARS-CoV-2 3CL^pro^.

### 3.3. The Luciferase Complementation Assay Can Easily Distinguish 3CL^pro^ Inhibition from Cytotoxicity 

**Figure 3:**
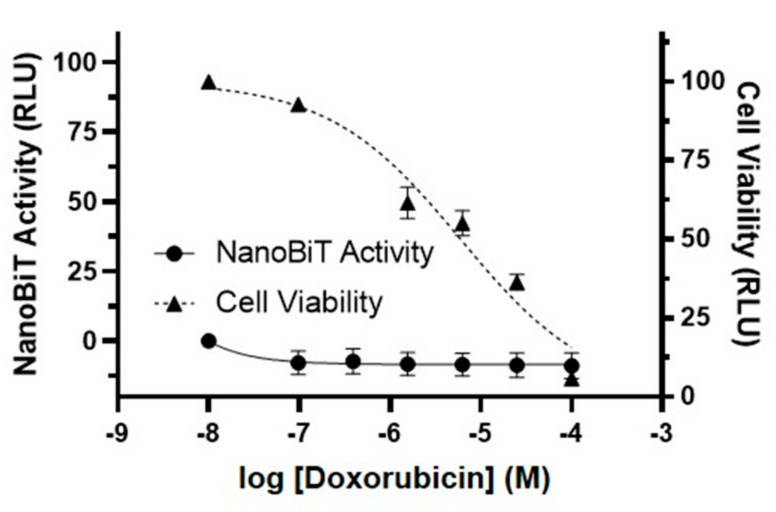
The luciferase complementation assay can easily distinguish 3CL^pro^ inhibition from cytotoxicity. To determine if the luciferase complementation reporter assay can differentiate 3CL^pro^ inhibition from cytotoxicity, 293T cells were transfected with the S-L-GFP lentiviral vector, and DMSO (control) or the anti-cancer drug doxorubicin (0.1–100 µM) was added. NanoBiT activity (circle, left axis) and cell viability (measured with the CellTiter-Glo 2.0 assay; triangle, right axis) were analyzed 30 h post-transfection and are expressed as relative luciferase units (RLU). Cell viability data were normalized to the DMSO sample, which was set to 100%. NanoBiT data were normalized to the DMSO and 100 µM GC376 samples, which were set to 0 and 100%, respectively. The data represent the mean ± standard deviation of three independent experiments.

In loss-of-function assays, cytotoxic compounds could be identified as hits, leading to false positives. In contrast, our luciferase complementation assay is a gain-of-function assay that should readily distinguish cytotoxic compounds from true inhibitors of SARS-CoV-2 3CL^pro^. To determine whether cytotoxicity could lead to false positives in our assay, we examined the impact of doxorubicin, a DNA topoisomerase II inhibitor and anti-cancer drug, on luciferase reporter activity and cell viability in 293T cells transfected with the S-L-GFP lentiviral vector (Figure 3). We found that doxorubicin resulted in a concentration-dependent decrease in cell viability, with a 50% cytotoxic concentration (CC_50_) of 3.7 µM. However, doxorubicin did not result in any significant change in luciferase reporter activity. These results clearly demonstrate that the luciferase complementation reporter assay can differentiate between 3CL^pro^ inhibition and cytotoxicity. This advantageous feature should eliminate many false positive hits caused by cytotoxic compounds when screening small molecules for activity against 3CL^pro^.

### 3.4. HIV Protease Inhibitors Do Not Block SARS-CoV-2 3CL^pro^ Activity in a Cell-Based Luciferase Complementation Assay

**Figure 4:**
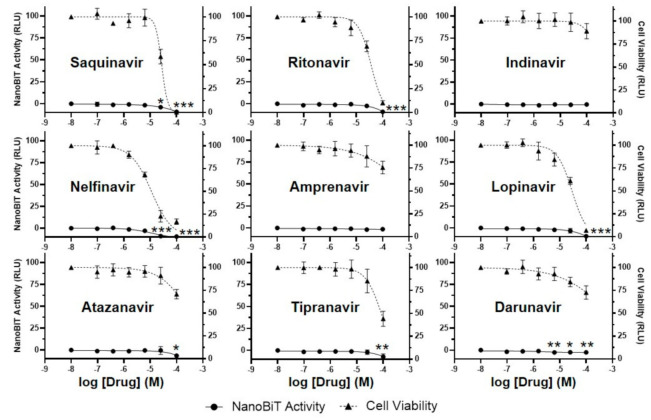
HIV protease inhibitors do not block SARS-CoV-2 3CL^pro^ activity in a cell-based luciferase complementation assay. To determine if HIV protease inhibitors are active against SARS-CoV-2 3CL^pro^ in cells, 293T cells were transfected with the S-L-GFP lentiviral vector, and DMSO (control) or inhibitors (0.1, 0.4, 1.6, 6.3, 25, or 100 µM) were added. NanoBiT activity (circle, left axis) and cell viability (measured with the CellTiter-Glo 2.0 assay; triangle, right axis) were analyzed 30 h post-transfection and are expressed as relative luciferase units (RLU). Cell viability data were normalized to the DMSO sample, which was set to 100%. NanoBiT data were normalized to the DMSO and 100 µM GC376 samples, which were set to 0 and 100%, respectively. The data represent the mean ± standard deviation of three independent experiments; * *p* < 0.05, ** *p* < 0.01, *** *p* < 0.001 (relative to no-drug; one-way ANOVA with Dunnett’s post-test).

Human immunodeficiency virus (HIV) protease inhibitors, particularly lopinavir and nelfinavir, have been reported to inhibit the replication of SARS-CoV-2 in cell culture [35,36,37]. However, other groups have reported that HIV protease inhibitors do not block SARS-CoV-2 3CL^pro^ in vitro, even at the maximal concentration tested (20 or 100 µM) [18,19,20]. In one recent report, the apparent anti-SARS-CoV-2 activity of lopinavir and nelfinavir in cell culture was found to be an artifact due to cytotoxicity [38]. Using our luciferase complementation reporter assay, we determined the effects of nine FDA-approved HIV protease inhibitors, including lopinavir and nelfinavir, on SARS-CoV-2 3CL^pro^ activity in cells. We transfected 293T cells with the S-L-GFP lentiviral vector, added DMSO (control) or varying concentrations of inhibitors, and measured luciferase activity and cell viability 30 h post-transfection. Results from three independent experiments showed that none of the HIV protease inhibitors resulted in significant enhancement of luciferase reporter activity (Figure 4, Table 1). Instead, for most of the inhibitors, there was a statistically significant decrease in luciferase activity at the highest concentration tested (100 µM), presumably due to cell death and loss of background luminescence. For indinavir and amprenavir, the least toxic inhibitors, there was no significant increase or decrease in luciferase activity. These results agree with previous findings from in vitro assays [18,19,20] and suggest that the possible inhibition of SARS-CoV-2 replication in cell culture by HIV protease inhibitors is due to cytotoxicity and/or off-target effects (see Discussion). These results also further demonstrate that our luciferase complementation assay can easily distinguish 3CL^pro^ inhibition from cytotoxicity.

### 3.5. Four Additional Compounds Inhibit SARS-CoV-2 3CL^pro^ in a Cell-Based Luciferase Complementation Assay: Boceprevir, Z-FA-FMK, Calpain Inhibitor XII, and GRL-0496

**Figure 5:**
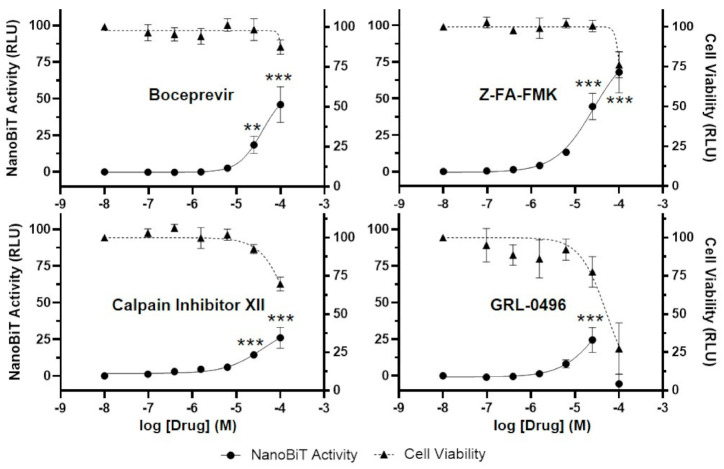
Boceprevir, Z-FA-FMK, calpain inhibitor XII, and GRL-0496, inhibit SARS-CoV-2 3CL^pro^ in a cell-based luciferase complementation assay. To determine if other compounds are active against SARS-CoV-2 3CL^pro^ in cells, 293T cells were transfected with the S-L-GFP lentiviral vector, and DMSO (control) or compounds (0.1, 0.4, 1.6, 6.3, 25, or 100 µM) were added. NanoBiT activity (circle, left axis) and cell viability (measured with the CellTiter-Glo 2.0 assay; triangle, right axis) were analyzed 30 h post-transfection and are expressed as relative luciferase units (RLU). Cell viability data were normalized to the DMSO sample, which was set to 100%. NanoBiT data were normalized to the DMSO and 100 µM GC376 samples, which were set to 0 and 100%, respectively. The data represent the mean ± standard deviation of three independent experiments; ** *p* < 0.01, *** *p* < 0.001 (relative to no-drug; one-way ANOVA with Dunnett’s post-test).

Although many inhibitors of SARS-CoV-2 3CL^pro^ have been reported in the literature, their ability to inhibit 3CL^pro^ in cells is uncertain due to issues such as cell permeability, cytotoxicity, and off-target effects. Using our newly developed luciferase complementation reporter assay, we determined the effects of 22 additional compounds on SARS-CoV-2 3CL^pro^ activity and cell viability. These compounds included five hepatitis C virus (HCV) protease inhibitors, a SARS-CoV 3CL^pro^ inhibitor (GRL-0496), three host/cellular cysteine protease inhibitors, and compounds of various other classes (Table 1). Most of these compounds have been reported to inhibit SARS-CoV-2 3CL^pro^ in vitro and possibly SARS-CoV-2 virus replication in cell culture. However, we also included several compounds that do not inhibit SARS-CoV-2 3CL^pro^ in vitro (telaprevir, asunaprevir, grazoprevir) [18,20] or that inhibit SARS-CoV-2 3CL^pro^ in vitro but not virus replication in cell culture (MAC 5576) [39]. Results from three independent experiments showed that, of the 22 compounds tested, only four clearly inhibited SARS-CoV-2 3CL^pro^ in our luciferase complementation reporter assay: boceprevir, Z-FA-FMK, calpain inhibitor XII, and GRL-0496 (Figure 5, Table 1). Of these, boceprevir, an FDA-approved HCV protease inhibitor, and Z-FA-FMK, originally developed as an inhibitor of cellular cysteine proteases and recently identified by high-throughput screening as an inhibitor of SARS-CoV-2 3CL^pro^ [24,40], had the greatest effect on 3CL^pro^ activity and little cytotoxicity. Calpain inhibitor XII, but not II, also inhibited SARS-CoV-2 3CL^pro^ in the cell-based luciferase assay, but to a lesser extent (Figure 5, Table 1). As the names imply, these compounds were originally developed as inhibitors of the calpain family of cellular cysteine proteases [41,42]. Calpain inhibitors II and XII have been proposed to have a dual mechanism of action against SARS-CoV-2 in cell culture: inhibition of 3CL^pro^ and inhibition of host proteases (such as calpains and cathepsin L) that are important for SARS-CoV-2 entry in certain cell types (see Discussion) [43]. GRL-0496 also inhibited SARS-CoV-2 3CL^pro^ in cells, but its activity and cytotoxicity were poorly separated, such that luciferase reporter activity actually decreased from 25 to 100 µM (Figure 5, Table 1). GRL-0496 was originally developed as an inhibitor of SARS-CoV 3CL^pro^ [44] but has also been found to be active against SARS-CoV-2 [45]. None of the remaining compounds had significant activity in our assay (Figure S1, Table 1), despite reported inhibition against SARS-CoV-2 3CL^pro^ in vitro and/or virus replication in cell culture. For these compounds, there are several possible explanations for the observed discrepancies, including poor cell permeability, lack of specificity, alternative mechanisms of action (i.e., off-target effects), and excessive cytotoxicity (see Discussion). 

## 4. Discussion

Although SARS-CoV-2 3CL^pro^ is widely considered an attractive target for antiviral drugs, the development of inhibitors has been impeded by a lack of cell-based assays that can be performed in a BSL-2 setting and are amenable to high-throughput screening. While many groups have performed in vitro screens using purified protein, prospective compounds often lack activity in cells due to poor permeability or high cytotoxicity. Alternatively, compounds can be assessed by examining their impact on SARS-CoV-2 replication in cell culture, but this approach requires a BSL-3 facility. Recently, a BSL-2 reporter system for identification of SARS-CoV-2 inhibitors in cells was developed [25]. In this assay, called Flip-GFP, 3CL^pro^-mediated cleavage leads to the formation of functional GFP, whereas inhibition of 3CL^pro^ leads to a loss of GFP fluorescence. One potential limitation of this approach is that it may be difficult to distinguish 3CLpro inhibition from cytotoxicity. Two additional cell-based reporter assays have been described in pre-print manuscripts. Resnick and co-workers have developed a system in which inhibitors are identified by their ability to prevent 3CL^pro^-mediated cytotoxicity, as measured by crystal violet staining [45]. While simple in principle, approaches based on measuring cytotoxicity could potentially identify compounds that inhibit 3CL^pro^-mediated toxicity through indirect effects, such as effects on gene expression or protein stability. Lastly, a gain-of-function approach has been described in which 3CL^pro^ and GFP expression are suppressed unless 3CL^pro^ is inhibited [55]. However, the mechanism of the assay has not been defined. 

Here, we have developed a novel luciferase complementation reporter assay that circumvents many of the limitations of previously described approaches. The assay is simple, rapid, and has a high signal-to-background ratio. As it represents a gain-of-function approach, the assay can easily distinguish inhibition of 3CL^pro^ from cytotoxicity. Using our assay, none of the FDA-approved HIV protease inhibitors had any effect on SARS-CoV-2 3CL^pro^ activity. These results agree with previous reports in which HIV protease inhibitors lacked detectable activity against 3CL^pro^ in vitro [18,19,20]. In contrast, other early studies found that some HIV protease inhibitors block SARS-CoV-2 replication in cell culture [35,36,37]. However, Hattori and co-workers recently demonstrated that these findings were likely false positives due to compound cytotoxicity [38]. In their study, lopinavir and nelfinavir initially appeared to have antiviral activity when SARS-CoV-2 replication, measured by qRT-PCR, was examined in Vero E6 cells. However, lopinavir and nelfinavir did not reduce the proportion of infected cells as determined by immunofluorescence microscopy, leading the authors to conclude that these compounds have no detectable antiviral activity. The combination of lopinavir and ritonavir has also been tested for COVID-19 in clinical trials, but no significant benefit has been observed [9,56,57]. Our findings reinforce the conclusions of Hattori and co-workers [38] and demonstrate the benefit of using a gain-of-function reporter assay to evaluate the antiviral activity of compounds. 

Using the luciferase complementation reporter assay, we confirmed the ability of the small molecule GC376 to inhibit SARS-CoV-2 3CL^pro^, as reported by others [18,19,20,34]. GC376 was developed as an inhibitor of another coronavirus, feline infectious peritonitis virus, but has broad activity against 3CL^pro^ from many coronaviruses [58]. In our assay, the EC_50_ of GC376 was slightly higher than those reported by others (Table 1). The cause of this is unclear, but it may be due to differences in experimental conditions (e.g., cell type, time of drug addition, or duration of drug treatment). We screened 22 additional compounds for anti-3CL^pro^ activity, including HCV protease inhibitors, cellular protease inhibitors, and various other compounds reported to inhibit SARS-CoV-2 3CL^pro^ in vitro and/or virus replication in cells (Table 1). Of these, only four compounds exhibited significant activity against 3CL^pro^ in cells: boceprevir, Z-FA-FMK, calpain inhibitor XII, and GRL-0496. Boceprevir is an FDA-approved HCV protease inhibitor reported by two groups to be active against SARS-CoV-2 3CL^pro^ [18,20]. Z-FA-FMK is a preclinical compound originally developed as an inhibitor of cellular cysteine proteases but recently identified as an inhibitor of SARS-CoV-2 3CL^pro^ by high-throughput screening [24,40]. Calpain inhibitor XII is another preclinical compound originally developed as an inhibitor of cellular cysteine proteases [41]. This compound has been proposed to have a dual antiviral mechanism of action: inhibition of 3CL^pro^ and inhibition of the host protease cathepsin L, which is important for SARS-CoV-2 entry in some cell types [43]. Lastly, GRL-0496 is a preclinical compound originally developed as an inhibitor of SARS-CoV 3CL^pro^ [44]. None of these compounds was able to inhibit SARS-CoV-2 3CL^pro^ to the same extent as GC376, however. All four compounds have been previously reported to block SARS-CoV-2 replication in cell culture [18,20,24,45].

For the 18 remaining compounds, we did not detect significant activity against SARS-CoV-2 3CL^pro^ in cells (Figure S1, Table 1). For the other HCV protease inhibitors, we did not expect to observe activity based on in vitro studies [18,20]. For the other compounds, there are several possible explanations for their lack of activity. First, the compounds may be too toxic, which would result in low luciferase activity regardless of whether they inhibited 3CL^pro^ in the luciferase reporter assay. For many of these compounds, their effect on cytotoxicity was not previously determined, or their cytotoxicity was poorly separated from their antiviral activity. Thus, it is not clear whether these compounds truly had activity against SARS-CoV-2 in cell culture. Our findings suggest that they do not, and that their possible antiviral activity may have been an artifact due to cytotoxicity, as described for lopinavir and nelfinavir [38]. Although most of these compounds were reported to inhibit 3CL^pro^ in vitro, they may have done so through non-specific mechanisms, as recently reported for ebselen [59]. Second, the compounds may not be toxic but still fail to inhibit 3CL^pro^ in cells due to poor permeability, metabolization into an inactive form, or a lack of specificity for 3CL^pro^. For example, MAC 5576 was reported to inhibit SARS-CoV-2 3CL^pro^ in vitro but had no effect on virus replication [39]. Third, some compounds may inhibit SARS-CoV-2 virus replication through a different mechanism of action, such as inhibition of PL^pro^ or cellular proteases. For example, calpain inhibitors II and XII have been reported to have a dual mechanism of action: inhibition of 3CL^pro^ and inhibition of the host protease cathepsin L [43]. Our findings suggest that the primary mechanism of action for calpain inhibitor II is blocking cathepsin L, whereas calpain inhibitor XII may primarily target 3CL^pro^. Alternative antiviral mechanisms of action have also been proposed for suramin and baicalein [51,52].

In sum, we have developed a novel luciferase complementation reporter assay for identification of SARS-CoV-2 3CL^pro^ inhibitors in living cells. The assay is sensitive, rapid, easy to perform, and can readily differentiate cytotoxicity from 3CL^pro^ inhibition, a powerful feature that should eliminate many false positives during screening. With minor modifications, this assay could potentially be used to identify inhibitors of 3CL^pro^ from other coronaviruses or inhibitors of other proteases that are considered targets for COVID-19 drug development. The widespread distribution of this reporter vector should significantly accelerate efforts to identify more potent 3CL^pro^ inhibitors.

## Figures and Tables

**Table 1:** Compounds screened for inhibition of SARS-CoV-2 3CL^pro^ in cells.

Compound	Class ^1^	Max Inhibition ^2^	EC_50_ (µM) ^3^	CC_50_ (µM) ^4^	Reported EC_50_ (µM) ^5^	Reported CC_50_ (µM) ^5^	References ^5^
GC376	FIPV PI	100%	23.8	>100	0.2–4.5	>100–>200	[18,19,20,34,39,43,46]
Saquinavir	HIV PI	NA	NA	27.3	8.8	44.4	[37]
Ritonavir	HIV PI	NA	NA	36.1	8.6	74.1	[37]
Indinavir	HIV PI	NA	NA	>100	59.1	>81	[37]
Nelfinavir	HIV PI	NA	NA	11.2	1.1–3.1	24.3–53.0	[37,38]
Amprenavir	HIV PI	NA	NA	>100	31.3	>81	[37]
Lopinavir	HIV PI	NA	NA	29.1	5.7–19.0	51.0–74.4	[37,38]
Atazanavir	HIV PI	NA	NA	>100	2.0–9.4	>81, 312	[36,37]
Tipranavir	HIV PI	NA	NA	85.1	13.3	76.8	[37]
Darunavir	HIV PI	NA	NA	>100	46.4, NA	>81–>100	[37,47]
Boceprevir	HCV PI	46%	38.6	>100	1.3–15.6	>100–>200	[18,20]
Telaprevir	HCV PI	NA	NA	71.5	ND	ND	-
Simeprevir	HCV PI	NA	NA	19.6	ND	ND	-
Asunaprevir	HCV PI	NA	NA	31.5	ND	ND	-
Grazoprevir	HCV PI	NA	NA	57.4	ND	ND	-
GRL-0496	SARS-CoV PI	24%	ND	53.2	9.1	81.0	[45]
Z-FA-FMK	Host PI	68%	26.3	>100	0.13	>20	[24]
Calpain Inhibitor XII	Host PI	26%	ND	>100	0.49	>100	[20]
Calpain Inhibitor II	Host PI	NA	NA	>100	2.1	>100	[20]
MG-115	Proteasome In	NA	NA	11.0	0.02	1.1	[24]
TBB	Kinase In	NA	NA	100	14.1	8.9	[24]
Masitinib	Kinase In	NA	NA	3.0	3.2	ND	[48]
DA-3003-1	Phosphatase In	NA	NA	7.7	4.5	7.7	[24]
Tafenoquine	Antiparasitic	NA	NA	2.0	2.5–15.7	37.2	[49,50]
Suramin	Antiparasitic	NA	NA	>100	20.0, NA	>20–>5000	[24,51]
Quinacrine	Antiparasitic	NA	NA	2.5	ND	ND	-
Walrycin B	Antibiotic	NA	NA	29.0	3.6	4.3	[24]
MK 0893	Miscellaneous	NA	NA	42.3	3.2	12.6	[24]
penta-O-galloyl-beta-D-glucose hydrate	Miscellaneous	NA	NA	30.4	12.6	11.2	[24]
MAC 5576	Miscellaneous	NA	NA	>100	NA	>100	[39]
Ebselen	Miscellaneous	NA	NA	8.0	4.7	ND	[21]
Baicalein	Miscellaneous	NA	NA	>100	1.7–17.6	>50–>200	[52,53,54]

^1^ Compound class: FIPV PI, feline infectious peritonitis virus protease inhibitor, HIV PI, human immunodeficiency virus protease inhibitor; HCV PI, hepatitis C virus protease inhibitor, SARS PI, severe acute respiratory syndrome coronavirus protease inhibitor; Host PI, host protease inhibitor; In, inhibitor. ^2^ Maximum inhibition of SARS-CoV-2 3CL^pro^ in cell-based luciferase complementation assay relative to 100 µM GC376, which was set to 100%; NA, no activity. ^3^ Half maximal effective concentration (EC_50_) values, as determined by the SARS-CoV-2 3CL_pro_ luciferase complementation assay. ND, not determined; EC_50_ values could not be calculated for two compounds due to weak activity (calpain inhibitor XII) or overlapping activity and cytotoxicity (GRL-0496); NA, no activity. ^4^ 50% cytotoxic concentration (CC_50_) values, as determined by the CellTiter-Glo 2.0 assay. ^5^ Previously reported EC_50_ (and corresponding CC_50_) values for inhibition of SARS-CoV-2 replication in cell culture. Note that, in some cases, EC_50_ values may be affected by compound toxicity. ND, not determined; NA, no activity.

## Data Availability

Data is contained within the article or supplementary material.

## Supplementary Materials

The following are available online at https://www.mdpi.com/1999-4915/13/2/173/s1, Figure S1: Compounds that lack activity against SARS-CoV-2 3CL^pro^ in a cell-based luciferase complementation assay.
