# Supplementary material for: Development of a Cell-Based Luciferase Complementation Assay for Identification of SARS-CoV-2 3CLpro Inhibitors
Source: Viruses. 2021 Jan 24;13(2):173. doi: 10.3390/v13020173 (PMC7911889; doi:10.3390/v13020173)
Supplement: Supplementary file 1 [file viruses-13-00173-s001.zip › viruses-1061357-supplementary.pptx]

## Slide 1
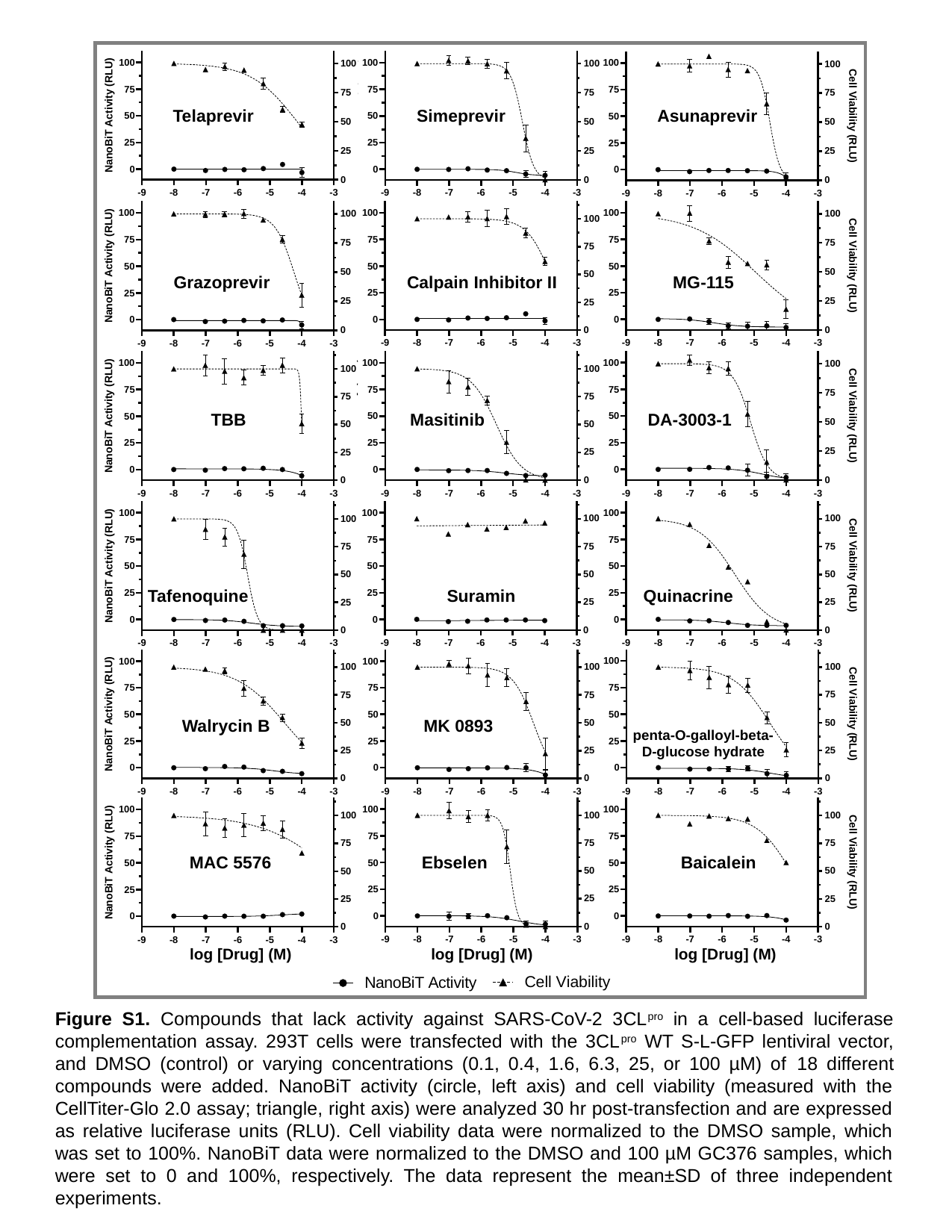

Telaprevir
Simeprevir
Asunaprevir
Grazoprevir
Calpain Inhibitor II
MG-115
TBB
Masitinib
DA-3003-1
Tafenoquine
Suramin
Quinacrine
Walrycin B
MK 0893
penta-O-galloyl-beta-D-glucose hydrate
MAC 5576
Ebselen
Baicalein
log [Drug] (M)
log [Drug] (M)
log [Drug] (M)
Figure S1. Compounds that lack activity against SARS-CoV-2 3CLpro in a cell-based luciferase complementation assay. 293T cells were transfected with the 3CLpro WT S-L-GFP lentiviral vector, and DMSO (control) or varying concentrations (0.1, 0.4, 1.6, 6.3, 25, or 100 µM) of 18 different compounds were added. NanoBiT activity (circle, left axis) and cell viability (measured with the CellTiter-Glo 2.0 assay; triangle, right axis) were analyzed 30 hr post-transfection and are expressed as relative luciferase units (RLU). Cell viability data were normalized to the DMSO sample, which was set to 100%. NanoBiT data were normalized to the DMSO and 100 µM GC376 samples, which were set to 0 and 100%, respectively. The data represent the mean±SD of three independent experiments.
